# Editorial: Novel Aspects of Neurotransmitters

**DOI:** 10.3389/fcell.2021.800765

**Published:** 2021-11-18

**Authors:** Z. G. Zhang, L. Pavon, H. Tu

**Affiliations:** ^1^ State Key Laboratory of Oncogenes and Related Genes, Shanghai Cancer Institute, Ren Ji Hospital, School of Medicine, Shanghai Jiao Tong University, Shanghai, China.; ^2^ Laboratorio de Psicoinmunología, Dirección de Investigaciones en Neurociencias del Instituto Nacional de Psiquiatría Ramón de la Fuente Muñiz, Ciudad de México, Mexico

**Keywords:** neurotransmitters, cancer, evolution, novel aspects, immunoregulation, asthma, anxiety and depression

Neurotransmitters are not the privilege of neural system and they have multiple functions in the peripheral organs. The aim of this special issue is to improve our understanding of non-canonical functions of neurotransmitters, as well as their novel aspects in neural system.

Cancer neuroscience is an emerging field and it reveals a promising future in preclinical and translational research. In this collection, there are several papers focus on the novel roles of neurotransmitters on cancer research.

Perineural invasion is a common phenomenon which indicates a poor prognosis in multiple cancers. Chen et al. made an interesting revision of perineural invasion and stress hormone in gynecological cancers (Chen et al.). More significantly, almost 100% pancreatic cancer has perineural invasion and neurotransmitters play important roles in the innate and adaptive immune responses in the pancreatic cancer microenvironment (Liang et al.).

We believe that cancer is a complicated, flexible, systemic disease encompassing multiple dysregulated processes within the neuro-endocrine-immune system, which might open a wide range of therapeutic options (Jiang et al.).

Besides in cancer, 5-Hydroxytryptamine, Glutamate, and ATP have broad biological functions on multiple types of non-neural cells (Franco et al.). Francelin et al. show in a systematized way about the participation of neurotransmitters in the maturation of T lymphocytes in the thymus (Francelin et al.). Neurotransmitters and neuropeptides are also involved in the pathological processes in asthma (Pavón-Romero et al.). In Anxiety and Depression, brain transmitters could be modulated by intestinal microbiota (Huang and Wu). The locus coeruleus (LC) tyrosine-hydroxylase (TH) neurons and the TH:LC-paraventricular thalamus circuit may be involved in regulating emergence from anesthesia (Ao et al., 2021).

Moroz leads us to explore, in an elegant dissertation, the evolutionary aspects of neurons origin. He presents the most relevant hypotheses on the aspects that have given rise to neuronal types, their organization, way of communication, and the mechanisms underlying these characteristics (Moroz). Moroz and Romanova present an interesting dissertation on the advantages of synapses in evolution in different evolutionary orders, discussing the pros and cons observed in developing this neural communication process, emphasizing the participation of lipid components and the involvement of organelles such as the endoplasmic reticulum and mitochondria (Moroz and Romanova).

Neurotransmitters are ancient molecules, e.g. acetylcholine exists in microorganisms (Whittaker, 1963). Most of neurotransmitters appeared earlier than the neural system. Besides in the neural system, neurotransmitters have a lot of basic functions to be discovered in the future, such as the serotonylation, which is a newly recognized post-translational modification (Muma and Mi, 2015; Bader, 2019) where serotonin is covalently incorporated into proteins via transamidation.

Therefore, we believe that the first step to take advantage of the full potential of the new aspects of neurotransmitters is to make them known and show their clinical, therapeutic, and research potential. With this research topic, we contribute to achieving this goal, and we are convinced that the readers of the papers that comprise it will acquire a comprehensive view of the full potential of this knowledge.
